# A Replication and Extension of Three Studies Investigating Escalation of Commitment and Regret Aversion

**DOI:** 10.1177/01461672251345021

**Published:** 2025-06-19

**Authors:** Nandita Dhanda, Ignazio Ziano, Kam Hoi Ching, Angela Kwun Lan Phuong, Yuen Ting Lo, Man Kin Yip, Bill Jiaxin Shi, Boley Cheng

**Affiliations:** 1 Geneva School of Economics and Management, University of Geneva, Switzerland; 2 Department of Psychology, University of Hong Kong, Hong Kong SAR, Hong Kong

**Keywords:** replication, escalation of commitment, sunk costs, regret aversion, decision making

## Abstract

This article presents preregistered replications of three influential studies in the field of escalation of commitment and regret aversion. In Study 1, we found partial support for Staw’s (1976) article on the effects of personal responsibility and consequences on the escalation of commitment. Study 2 failed to replicate Zeelenberg et al. (1996) article examining the role of feedback on risk preferences. The extension of study 2 found a small correlation between the tendency to experience regret and risk preferences of participants in the Safe ONLY condition. In Study 3, we failed to replicate Wong and Kwong’s (2007) results that showed a causal effect of personal responsibility and regret possibility on the escalation of commitment. Moreover, study 3’s extension found no support for individual differences in neuroticism on the escalation of commitment. The impact of this replication on our understanding of decision-making processes and the factors that contribute to individuals’ commitment to their choices will be discussed.

The role of regret aversion and escalation of commitment^1^ on decision making has been extensively studied in the fields of social psychology (Zhang & Baumeister, 2006), finance (Schulz & Cheng, 2002), and economics (Camerer & Weber, 1999). Escalation of commitment is defined as continued commitment to an initial choice of action, despite the increasingly negative outcomes associated with it. In this article, we replicated three prominent studies of escalation of commitment and its psychological drivers, specifically anticipated regret, regret aversion, and personal responsibility. Study 1 is a very close replication of Staw’s (1976) article examining the effect of personal responsibility and consequences on the escalation of commitment. Study 2 is a very close replication and extension of Zeelenberg et al.’s (1996) study looking at the role of regret aversion as a motivator in escalation of commitment. Lastly, in study 3, we report a close replication and extension of studies 1a and 2a of Wong and Kwong’s (2007) article examining the role of personal responsibility and regret possibility on the escalation of commitment. We further examine the influence of individual differences in neuroticism on escalation of commitment.

## Escalation of Commitment

Research has indicated the tendency of individuals to remain committed to their initial choice, even in the light of unsuccessful resulting outcomes (Arkes & Blumer, 1985; Staw, 1976; Thaler, 1980). This phenomenon termed as escalation of commitment (Staw, 1976) is marked with three defining properties (Bowen, 1987; Staw, 1982). First, a series of behavior linked by an initial choice to achieve a goal state. This includes resources (such as money, time, or effort) being invested in the initial choice. Second, negative feedback regarding the effectiveness of the initial actions in achieving the goal state. Third, this creates an opportunity for the decision-maker to decide whether to continue investing in (in order to recover the previous costs) or withdraw from the initial choice. In this two-option situation, escalation of commitment by the decision-maker is marked by pursuing the losing course of action (Brockner, 1992; Staw & Ross, 1987).

Research has examined psychological drivers of escalation of commitment which include the need to justify or commit to the initial decision (Arkes & Blumer, 1985; Ku, 2008), personal responsibility for prior decisions (Staw, 1976), as well as regret (Zeelenberg & Beattie, 1997). Regret and regret aversion are central to understanding the escalation of commitment. Based on regret theory, people tend to avoid choosing options that led to regret (Bell, 1982; Loomes & Sugden, 1982). In other words, individuals actively try to minimize the possibility of regret in their decision making (Zeelenberg & Beattie, 1997). Under escalation situations, regret stems from the belief that changing previous decisions would have resulted in better outcomes (Landman, 1994; Zeelenberg & Pieters, 2007) and this regret is further exacerbated when individuals learn that the potential outcome of an alternative decision is likely to be more favorable than the one they chose (Zeelenberg et al., 1996). In addition to experiencing regret retrospectively (i.e., by examining the factors or decisions that occurred before the decision), research has further adopted a forward-looking approach by examining the regret a decision-maker would experience if they made a particular decision, that is, anticipated regret (Simonson, 1992; Zeelenberg et al., 1996).

Empirical evidence has been found on this regret-aversive state in different contexts such as before deciding on a big investment (Wong & Kwong, 2007), in business markets (Sarangee et al., 2019), or after experiencing losses over a choice and given the alternative to either continue investing or withdrawing (Zeelenberg & Beattie, 1997).

## Choosing Studies to Replicate

We selected these three studies for replication due to three broad factors: a comprehensive understanding of escalation of commitment, the absence of direct replications, and impact. First, as influential studies in decision making and escalation of commitment and considering the significant impact of these articles on scholarly research in social psychology, judgment and decision making, and behavioral economics, it is important to replicate these findings. This is reflected, for example, in the number of citations for these studies with Staw (1976), Wong and Kwong (2007), and Zeelenberg et al. (1996) having 3,007, 219, and 706 citations, respectively. Importantly, at the time of writing this article, it has been almost 50 years since Staw (1976) was published. Thus, by replicating this effect, we can examine whether the findings from Staw (1976) stood the test of time.

Second, to the best of our knowledge, there are no published “close” or “very close” replications for Wong and Kwong (2007) and Zeelenberg et al. (1996) thus far (see Table 1 for classification of each of our replications according to LeBel et al. (2017)). Staw (1976), however, has been unsuccessfully replicated with Polish-speaking participants (Białek et al., 2021). Previous research has indicated cultural differences in escalation behavior (Keil et al., 2000; Liang et al., 2014) and thus, it is important to replicate Staw’s (1976) findings in different cultural contexts to understand whether escalation tendencies are driven by universal psychological mechanisms such as self-justification bias. Moreover, escalation of commitment is a phenomenon that is marked with important practical implications. Escalation of commitment can occur in daily life in situations such as causal betting between friends and real-life financial decisions. Examples can further include staying in a job that one dislikes and sticking to unproductive business strategies or a failing project.

**Table 1.** Replication Classification Based on LeBel et al. (2017).

Design facet	Staw (1976)	Zeelenberg et al. (1996)	Wong and Kwong (2007)
Independent Variable operationalization	Same	Same	Same
Dependent Variable operationalization	Same	Same	Same
IV stimuli	Same	Same	Different
DV stimuli	Same	Same	Different
Procedural details	Different	Different	Different
Physical setting	Different	Different	Different
Contextual variables	Different	Different	Different
Replication classification	Very close replication	Very close replication	Close replication

With regard to their impact, taken together, these studies provide a comprehensive framework to understand escalation of commitment by explaining the cognitive, emotional, and motivational factors that drive individuals to remain committed to failing courses of action. From a cognitive perspective, as suggested by Staw (1976), escalation of commitment is explained through the self-justification bias that leads individuals to rationalize their past decisions and overlook negative consequences of their actions to maintain consistency. Zeelenberg et al. (1996) suggested the role of emotions associated with the fear of anticipated regret influencing individual’s commitment. From a motivational perspective, as indicated by the findings of Wong and Kwong (2007), anticipated regret motivates individuals to stay committed to their initial choice. In the following paragraphs, we introduce the selected studies and the effects we are replicating, and we discuss the importance of replicating these effects to further our understanding of escalation of commitment (refer to Table 10 for the effect sizes of the target articles).

Our study 1 replicated Staw’s (1976) study that examined the effect of responsibility, consequence, and their interaction on escalation of commitment. This study examines the cognitive factors driving escalation of commitment by adopting a retrospective lens and focusing on factors that occur before the escalation decisions. From a cognitive perspective, escalated commitment can be explained by self-justification bias. Through escalated commitment to their prior decision, the self-justification bias allows individuals to maintain their self-image by avoiding to admit the negative outcomes associated with their decision. Based on the original study, we expect higher commitment when individuals are personally responsible for an initial failing decision. Additionally, the original study found support for the effect of the interaction of high personal responsibility and negative consequences on commitment to prior financial decisions. These findings are essential to replicate, as following Staw (1976), research studying the escalation of commitment adopted a retrospective perspective by focusing on factors that occurred before the escalation decisions. These include examining the role of initial responsibility for the decision (Conlon & Parks, 1987), sunk costs (Arkes & Blumer, 1985; Garland, 1990), as well as financial (Tan & Yates, 2002) and mental (Heath, 1995) budgets in escalation decisions.

In our study 2, we examine the role of emotional factors in understanding escalation of commitment by replicating and extending Zeelenberg et al. (1996) study investigating the role of regret aversion as a motivator in decision-making. The original study suggested that the effect of regret aversion is stronger than that of risk aversion on decision-making in risky situations, and it was hypothesized that people will choose regret-minimizing options, which may or may not be risk-minimizing. Based on this, self-justification (as suggested by the findings of Staw (1976)) might not be the only driver of commitment to prior decisions and fear of regret might make people persist in their initial choice in risky situations. It is important to replicate this finding in a well-powered study considering its generalizability to real-life financial decisions. Evidence along these lines has been found across different experimental decision situations (Reb, 2008) as well as real-life lottery participation decisions (Zeelenberg & Pieters, 2004). Zeelenberg et al. (1996) suggested that people make regret-minimizing choices, which depending on the situation, can either be risk-averse or risk-seeking. Driven by self-justification (Staw, 1976) and avoiding regret (Larrick & Boles, 1995), risk-seeking individuals might persist longer in failing endeavors, whereas risk-averse individuals might not escalate due to the fear of worsening regret by continued investment in a failing decision (Wong & Kwong, 2007). Therefore, we added an extension to this study, examining individual differences in risk-preference of participants based on their tendency to experience regret.

Our study 3 aimed to replicate and extend Wong and Kwong’s (2007) studies 1a and 2a, examining the effects of personal responsibility and regret possibility on escalation of commitment. Contrary to the existing literature at the time that studied escalation of commitment from a retrospective lens (see Arkes & Blumer, 1985; Staw, 1976), Wong and Kwong (2007) adopted a prospective lens by focusing on the factors that occur after the escalation decisions. Combining the retrospective and prospective lens, this study provided a motivational explanation by examining the role of personal responsibility (i.e., retrospective lens) and anticipated regret (i.e., prospective lens) as drivers of escalation of commitment. Based on the original study, higher escalation of commitment is expected when personal responsibility and regret possibilities are higher. Previous research has found individual differences in personality traits as another determinant of escalation of commitment (Moon et al., 2003; Sleesman et al., 2012; Wong et al., 2006). Wong et al. (2006) explored the role of neuroticism and found that people with higher levels of neuroticism tend to escalate commitment less. Additionally, research has found that anticipated regret and personal responsibility influence escalation tendencies of individuals with high levels of neuroticism (Wong et al., 2006), and a higher tendency to anticipate regret in decision makers with high neuroticism (Moore & McElroy, 2012). Therefore, we added an extension to examine the influence of individual differences in neuroticism on the escalation of commitment.

Lastly, it is essential to replicate these effects as the latest years have been marked with a growing recognition of transparency, open science practices, and replicability in psychological science (Brandt et al., 2014; Nosek & Lakens, 2014; Open Science Framework, 2015). In light of this replication crisis faced by psychological research, replicating these studies will test whether the effects of these decision-making biases (i.e., regret aversion and self-justification) used to explain escalation of commitment are stable over time and context. These replications are a part of an ongoing mass-scale project focused on replicating classic findings from social psychology and judgment and decision-making (CORE Team, 2024).

## Transparency and Openness

All studies, measures, manipulations, and data/participant exclusions are reported in the manuscript or its Supplementary Material. All data, analyses, materials, and pre-registrations for the present article are available at https://osf.io/v9djc/?view_only=1c2ab5a4faec4002a2944f4024ed1e85.

## Replication Evaluation

## Study 1: Replication of Staw (1976)

In his classic study, Staw (1976) found empirical evidence for self-justification as a driver of escalation of commitment by showing higher commitment to a prior decision with negative outcomes (i.e., negative consequences) and for which the individual was personally responsible (i.e., high personal responsibility). The original study adopted a 2 (personal responsibility: high vs. low) × 2 (consequences: positive vs. negative) design and was conducted on a sample of 240 undergraduate students from the University of Illinois, Urbana Champaign. In the original study, participants were asked to complete an experiment in which they made hypothetical financial decisions for a company.

### Methods

The study was preregistered at https://osf.io/fm8h4/?view_only=665088d989624a7bb27a1ce8e4078185

#### Participants

A G*Power analysis using the effect sizes of the original study (*d* = 0.49 for personal responsibility and *d* = 0.55 for consequence) at 95% power and α = .05 suggested a sample size of 219. The replication study included 403 participants (recruited via Amazon MTurk in November 2018) to account for non-completion rates and maintain data quality. Three participants were excluded from the analysis due to incomplete returns and the final analysis included 400 participants (*M*_age_ *=* 39.09, *SD*_age_ = 11.48 years, 208 females, 192 males).

#### Design and Procedure

The present study is a “very close replication” of Staw’s (1976) experiment (see Table 1) and adopts identical procedures and materials. The experiment adopts a 2 × 2 experimental design with personal responsibility (high vs. low) and consequences (positive vs. negative) as the two independent variables. The variable consequence is manipulated on two aspects: First Choice and Financial Data, each having two levels: Consumer Division and Industrial Division. These aspects determine whether the first choice was the consumer or industrial division, and whether the financial information provided favored the consumer or industrial division. Table 2 contains a summary of the six experimental conditions to which participants are randomly assigned.

**Table 2.** Summary of the Six Experiment Conditions Based on Responsibility, First Choice, and Financial Data in Staw (1976).

Personal responsibility	Consequence	Financial data	
	First choice	Consumer > industrial (assigned)	Industrial > consumer (assigned)
High	Made by a participant	1. Data favors consumer	2. Data favors industrial
Low	First choice consumer (assigned)	3. First choice consumer, data favors consumer (positive consequence)	5. First choice consumer, data favors industrial (negative consequence)
	First choice industrial (assigned)	4. First choice industrial, data favors consumer (negative consequence)	6. First choice industrial, data favors industrial (positive consequence)

As in the original study, participants were asked to complete a financial decision-making experiment for a hypothetical company. The scenario presented to the participants included a case description for a hypothetical company at a financial decision, called D1. At D1, 10 million dollars were allocated to one of the two company divisions (Consumer division or Industrial division). Following this allocation, participants were provided information regarding the financial consequences of this decision (D1) over the last 5 years and were asked to make another financial decision (called D2) for the company. For D2, participants were asked to allocate 20 million dollars between the two divisions of the company. Information provided to the participants (refer to Table 3) differed based on the two independent variables (i.e., personal responsibility and consequences).

**Table 3.** Summary of Choices Made by Participants at Financial Decision D1 and D2 Based on 2 × 2 IV (i.e., Personal Responsibility and Consequence) Conditions in Staw (1976).

Conditions	D1	D2 (choice at D2 determines the commitment to prior financial decision at D1)
High personal responsibility—positive consequence	Participant makes the choice	Financial data following D1 *favors* the participant’s choice at D1
Low personal responsibility—positive consequence	Participant is assigned to a choice made by another financial officer, which either favors the consumer or industrial division	Financial data following D1 *favors* the assigned choice at D1
High personal responsibility—negative consequence	Participant makes the choice	Financial data following D1 does *not* favor the participant’s choice at D1
Low personal responsibility—negative consequence	Participant is assigned to a choice made by another financial officer which either favors the consumer or industrial division	Financial data following D1 does *not* favor the assigned choice at D1

In the high personal responsibility condition, both financial decisions at D1 and D2 were made by the participants. Participants in the low personal responsibility condition were only asked to make an allocation at D2 and were provided information about the financial decision at D1, which was made by another financial officer.

Decision consequences were experimentally manipulated through financial information provided to the participants. In the positive consequence condition, participants received financial information that showed that their chosen division at D1 had returned to profitable levels while the unchosen division continued to decline. Participants in the negative consequence condition, on the other hand, received data that showed a deepening decline in profitability of their chosen division but an improvement in the unchosen division. This resulted in two experimental conditions for the high personal responsibility condition and four experimental conditions for the low personal responsibility condition (refer to Table 2).

The materials used, including case descriptions and the financial data follow closely or are the same as the original study. The financial consequence data of D1 (Consumer >  Industrial; Industrial > Consumer) are the same sets of numerical values, differing in the labeling of Consumer or Industrial division as being the best investment.

#### Measures

Commitment to prior financial decisions was measured through the size of financial allocation made by the participants at D2 to the division chosen at D1. Participants were asked to indicate the amount of funds they would allocate to the two divisions, ranging from 0 to 20 million dollars. Following this decision, participants were asked to give a brief explanation for their choice.

### Results

The final analysis was conducted on a sample of 400 participants. Tables 4 and 5 show the allocations made by the participants in each condition.

**Table 4.** Mean and Standard Deviation of Allocation to Prior Choice (DV) Organized According to 2 (Responsibility: High vs. Low) × 2 (Consequence: Positive vs. Negative) Design in the Replication of Staw (1976).

	High responsibility		Low responsibility	
	*M* ^a^	*SD* ^a^	*M* ^a^	*SD* ^a^
Positive consequence	11.49	4.70	9.02	4.56
Negative consequence	8.41	5.04	6.99	4.89

a The values are in millions.

**Table 5.** Mean of Allocation to Prior Choice (DV) Organized According to 2 (Responsibility: High vs. Low) × 2 (First Choice: Consumer vs. Industrial) × 2 (Financial Data: Consumer > Industrial vs. Industrial > Consumer) experiment cases^a^ in the replication of Staw (1976).

Personal responsibility	Consequence	Financial data			
	First choice	Consumer > Industrial		Industrial > Consumer	
		*M*	*SD*	*M*	*SD*
High	First choice consumer	11.83 (Pc)*	4.05 (Pc)	7.75 (Nc)	4.02 (Nc)
	First choice industrial	8.73 (Nc)	5.47 (Nc)	11.3 (Pc)	5.03 (Pc)
Low	First choice consumer	9.42 (Pc)	5.15 (Pc)	7.63 (Nc)	5.56 (Nc)
	First choice industrial	6.35 (Nc)	4.05 (Nc)	8.63 (Pc)	3.93 (Pc)

* Pc and Nc refer to Positive and Negative consequence respectively.

a In addition to the six experimental conditions that participants were randomly assigned to, they further differ across two more conditions which depends on the decision made by the participants in the high personal responsibility condition.

A 2 × 2 ANOVA was employed to determine the differences in commitment to financial decision based on personal responsibility (high vs. low) and consequences (positive vs. negative) indicated support for an effect of personal responsibility (*F*(1,396) = 16.39, *p* < .001, η_p_^2^ = 0.040, 90% CI [0.014, 0.076]) and consequence (*F*(1,396) = 28.25, *p* < .001, η_p_^2^ = 0.067, [0.032, 0.109]) on commitment. However, we found no support for an interaction of these terms on commitment (*F*(1,396) = 1.20, *p* = .273, η_p_^2^ = 0.003, [0, 0.019]). Tukey’s post hoc test indicated pairwise differences in personal responsibility and consequence conditions. Participants in high personal responsibility conditions were found to be more committed to the prior financial decision as compared to the participants in the low personal responsibility conditions (*t*(396) = 4.05, *p* < .001, *d* = 0.41 [0.21, 0.60]). Similarly, participants in the positive consequence conditions were found to be more committed to the prior financial decision as compared to the participants in the negative consequence conditions (*t*(396) = 5.32, *p* < 001, *d* = 0.53 [0.33, 0.73]). This is further revealed in the mean differences in allocations made by the participants in different conditions (Table 4).

These findings are partially consistent with the original study which found support for the effect of personal responsibility (High > Low, *F*(1,235) = 14.40, *p* < .001, η_p_^2^ = 0.057, 90% CI [0.019, 0.112]) and consequences (Negative >  Positive, *F*(1,235) = 17.93, *p* < 001, η_p_^2^ = 0.071, [0.027, 0.128]) on commitment. For consequences, an effect was established, but in the opposite direction as the positive consequence condition was found to lead to significantly higher commitment than the negative consequence condition. Additionally, the original study found support for the effect of the interaction of personal responsibility and consequence (High responsibility—Negative consequence, *F*(1,235) = 5.56, *p* < .019^2^, η_p_^2^ = 0.023, [0.002, 0.064]). These are inconsistent with our findings, which found the High responsibility—Positive consequence as the highest significant interaction terms.

The post hoc analysis further suggested support for differences in the interaction conditions. Participants in the high personal responsibility—positive consequence condition were found to be more committed to the prior financial decision compared to the high personal responsibility—negative consequence condition (*t*(396) = 4.54, *p* < .001, *d* = 0.64, 95% CI [0.36, 0.92]), low personal responsibility—positive consequence condition (*t*(396) = 3.65, *p* < .05, *d* = 0.52, [0.24, 0.8]), and low personal responsibility—negative consequence condition (*t*(396) = 6.71, *p* < .001, *d* = 0.94, [0.65, 1.22]). The participants in low personal responsibility—positive consequence were more committed to the prior financial decision compared to the low personal responsibility—negative consequence condition (*t*(396) = 2.98, *p* < .05, *d* = .42, [0.14, 0.70]). However, it should be noted that our data violates the assumption of normality (*W* = 0.99, *p* < .001).

Independent samples *t*-test indicated no support for differences in commitment to the prior financial decision based on first choice (*t*(398) = 0.28, *p* = .78, *d* = 0.03, 95% CI [−0.17, 0.23]) and financial data (*t*(398) = −0.55, *p* = .58, *d* = −0.06, [−0.25, 0.14]). This indicates no significant differences exist whether the money was allocated to the consumer division (*M* = 9.07, *SD* = 5.10) or industrial division (*M* = 8.93, *SD* = 5.04) at the first financial decision D1. Furthermore, for financial data, no significant differences exist whether fund allocation was c > i (*M* = 8.85, *SD* = 5.12) or i > c (*M* = 9.13, *SD* = 5.00) at the first financial decision D1.

### Discussion

In this study, we attempted to replicate Staw’s (1976) original article, which found high personal responsibility and negative consequences lead to higher escalation to commitment. With their original study, Staw’s (1976) examined the role of retrospective factors (i.e., personal responsibility and consequences) on the escalation of commitment. These findings suggested the role of self-justification bias in rationalizing one’s past decisions associated with negative outcomes. The replication was partially successful. Similar to the original study, we found support for the effect of personal responsibility (High > Low) and consequences (Positive > Negative) on escalation to commitment. These findings are partially consistent with the original study, as we found an effect of consequences in the opposite direction compared to the original study. Moreover, unlike the original study, we found no support for interaction effects of personal responsibility and consequences on escalation to commitment. It is important to know the results of this replication primarily because Staw (1976) is a key article in the literature on escalation of commitment, examining cognitive factors driving escalation behavior. Previous attempts to replicate in Polish-speaking participants have been unsuccessful (Białek et al., 2021) which might be attributed to procedural differences as the study materials were translated to Polish. Taken together, these findings suggest that self-justification might not be a universal bias, and thus, future studies should examine the role of other cognitive factors in explaining the escalation of commitment.

## Study 2: Replication and Extension of Experiment 1 of Zeelenberg et al. (1996)

This study is key as it challenged existing work at the time which suggested that anticipated regret leads to risk-aversion (see Simonson, 1992; Josephs et al., 1992; Richard et al., 1996). By examining the influence of anticipated regret in risky decision-making, the authors proposed that people make regret-minimizing choices, which, depending on the situation, can either be risk-averse or risk-seeking. The original study was conducted on a sample of 76 undergraduate students from the University of Amsterdam, and participants were randomly assigned to one of the three feedback conditions.

### Methods

This study was pre-registered at https://osf.io/y8eah/?view_only=1c1df00e8feb427bb5f7b3f993077f02.

#### Participants

A G*Power analysis using the effect size (*d* = 0.61) of the original study at 95% power and α = .05 suggested a sample size of 376 participants (Faul et al., 2007). The present study recruited 452 participants using Amazon MTurk (to account for exclusion and non-completion rates) in November 2018. The study included four pre-registered exclusion criteria which can be found in the Supplemental Material at p. 8. Eight participants were excluded from analyses as they failed to provide a value for *X* (one of the measurements we asked) in the first phase of the experiment and 3 participants were excluded as they indicated they were not serious about filling the survey, which left a final sample of 441 participants (*M*_age_ = 39.67, *SD*_age_ = 11.68, 233 females, 208 males).

#### Design and Procedure

The present replication was conducted in two phases as the original study and used the same measures. Participants were randomly assigned to one of the three feedback conditions: Both Risky/safe, Risky only, and Safe only. In each condition, participants chose between a risky gamble (Gamble R) and a safe gamble (Gamble S). The risky Gamble R was associated with a higher payoff and a lower probability of winning and was riskier than the safe Gamble S, which had a higher probability of winning (refer to Table 7). In both Risky/Safe conditions, participants learned that they would only know the outcome of the chosen gamble. In the Risky ONLY condition, participants learned that they would always know the outcome of the risky option (Gamble R), and that they would only know the outcome of the safe option (Gamble S) if they chose it. In the Safe ONLY condition, participants learned that they would always know the outcome of the safe option (Gamble S), and they would only know the outcome of the risky option (Gamble R) if they choose it. As in the original study, Gamble R was presented as Gamble A and Gamble S was presented as Gamble B in the survey. This was done to avoid demand effects and in order to not influence participant’s perception of riskiness of the gambles. For clarity, we will use the terms Gambles R and S in this manuscript. See Table 6 for a detailed summary of the feedback in each condition.

**Table 6.** Summary of Feedback Provided to Participants in the Three Conditions in Zeelenberg et al. (1996).

Condition	Gamble chosen	Feedback obtained
Choice only Feedback (BOTH Risky/safe)	Risky (Gamble R)	Risky only
	Safe (Gamble S)	Safe only
Risky Feedback (Risky ONLY)	Risky (Gamble R)	Risky only
	Safe (Gamble S)	Both
Safe Feedback (Safe ONLY)	Risky (Gamble R)	Both
	Safe (Gamble S)	Safe only

Phase 1 of the study included matching where participants were presented with two hypothetical Gambles: Gamble R and Gamble S. Participants were told that each gamble could lead to a positive outcome, but Gamble R (with higher payoff and lower probability of winning) was riskier than Gamble S. The highest amount that could be won via Gamble R was $75. See Table 7 below for a summary of the gamble payoffs.

**Table 7.** Summary Table on the Gamble Payoffs in Zeelenberg et al. (1996).

Gamble R (Risky option)	Gamble S (Safe option)
$75 with 35% probability	$*X* with 65% probability
$0 with 65% probability	$0 with 35% probability

Then, participants were asked to assign a value of *X* as well as rate the attractiveness of the gambles. Four comprehension checks were added in this phase of the study to ensure that the participants understood the payoffs in each gamble.

The independent variable for this study was the feedback available to the participants (3 levels: Safe ONLY, Risky ONLY, BOTH Risky/Safe). In phase 2, participants in each condition explicitly learned about the possible feedback on foregone outcomes (see Table 6). Then, they were asked to select a Gamble (risky or safe). They were further asked to state the strength of their preference for the chosen gamble and respond to the Choice Regret Scale (described below).

#### Measures

Participants were asked to assign a value to *X* that makes the safe gamble (Gamble S) exactly as attractive as the risky gamble (Gamble R), and to rate the attractiveness of each gamble using a 1-item measure responded on a 12-point Likert scale (anchored at 1 = *not attractive* and 12 = *very attractive*). This was done to check whether the matching procedure produced equally attractive gambles as well as to determine the absolute level of attractiveness for each gamble. Participants were asked to select a gamble of choice (i.e., risky or safe).

Participants were asked to state their strength of preference for the chosen gamble on a 12-point Likert scale (anchored at 1 = *weak preference* and 12 = *strong preference*). Then, participants were further asked to write down the reasons for choosing a particular gamble. This was done to explore how participants justify their choices (refer to the Supplemental Material at p. 8 for the results).

Participants also completed the Choice Regret Scale (Schwartz et al., 2002), a 5-item measure responded on a 7-point Likert scale (anchored at 1 = *completely disagree* and 7 = *completely agree*) to assess their tendency to experience regret. Higher scores on the choice regret scale indicated higher tendencies to experience regret.

### Results

#### Value of *X*

First, a one-way ANOVA was used to determine any differences in the assigned values of *X*. The findings indicated support for differences in the assigned value of *X* across the three conditions, *M*_Risky ONLY_ = 49.8 (*SD* = 25.35), *M*_Safe ONLY_ = 56.4 (*SD* = 27.01), *M*_BOTH Risky/Safe_ = 47.7 (*SD* = 23.72), *F*(2,438) = 4.75, *p* < .05, η_p_² = .021, 90% CI [0.003, 0.046]. These results are inconsistent with the original study which reported no support for differences in the value assigned to *X* across the three experimental conditions (i.e., no significant differences in the payoff for Gamble S that makes it exactly as attractive as Gamble R). Tukey post hoc analysis indicated higher value being assigned to the safe gamble to make it as attractive as the risky gamble in the safe ONLY condition compared to the BOTH risky/safe condition, *t* = 8.75, *p* < .01.

#### Gamble Attractiveness

Consistent with the original study, a paired-samples *t*-test found support for difference in the levels of attractiveness for the two gambles *t*(440) = 12.98, *p* < .001, *d* = 0.62, 95% CI [0.52, 0.72] with Gamble S (*M* = 8.08, *SD* = 2.58) reported as being more attractive than Gamble R (*M* = 5.64, *SD* = 2.70).

#### Gamble Choice

A chi-square test of association indicated no support for differences in choices between the participants across the three feedback conditions, χ^2^(2) = 1.48, *p* = .476, *V* = 0.058. Most participants chose Gamble S (79%) regardless of which feedback condition they were assigned to. These findings are inconsistent with the original study which found a difference in choice frequency across the three feedback conditions, χ^2^(2) = 7.88, *p* < .02, *d* = 0.67 [0.19, 1.14], with more participants choosing Gamble R (the more risk-seeking choice) in Risky ONLY condition than the participants in the Safe ONLY condition. Therefore, we found no support for the original hypothesis that expected feedback promotes risk-aversion and risk-seeking in our replication.

#### Strength of Risk Preferences

Next, to determine the strength of risk-seeking by the participants, a new variable (risk preference) was calculated, as in the original paper. Risk preference ranged from −11.5 (*extreme risk aversion*) to 11.5 (*extreme risk-seeking*) and was calculated by using the formula: (risk preferences = choice (Gamble R was recoded as 1 and Gamble S was recoded as −1) × [strength of preference −1/2]), as in the original article.

A one-way ANOVA indicated no support for differences in the participants’ strength of risk preference across the three conditions, *F*(2,438) = 1.09, *p* = .337, η_p_² = 0.005, 90% CI [0, 0.018]. These findings are inconsistent with those of the original study, which found support for differences in the strength of risk preferences among participants of the three conditions, *F*(2,75) = 3.46, *p* < .05, η_p_² = 0.084, [0, 0.20]. The original study found that participants in Risky ONLY had a higher risk preference than of participants in Safe ONLY and BOTH Risky/Safe. On the other hand, participants in the three conditions in our study had low risk preferences, as reflected by the negative mean values (*M*_Risky ONLY_ = −5.85 [*SD* = 6.05]; *M*_Safe ONLY_ = −4.93 [*SD* = 7.19]; *M*_BOTH Risky/safe_ = −4.75 [*SD* = 7.10]).

#### Extension

An ANCOVA with strength of risk preference as a covariate suggested no support for differences in the tendency to experience regret in participants across the three conditions, *F*(2,437) = 0.36, *p* = .697, η_p_² = 0.002, 90% CI [0, 0.009]. Descriptive statistics indicate that participants in the three conditions have a moderate tendency to experience regret, *M*_Risky ONLY_ = 4.18 (*SD* = 0.95); *M*_Safe ONLY_ = 4.27 (*SD* = 0.92); *M*_BOTH Risky/Safe_ = 4.21 (*SD* = 0.86).

Lastly, the association of the tendency to experience regret and risk preference of participants in the three conditions was explored. Findings indicated a small correlation between the tendency to experience regret and risk preferences of participants in Safe ONLY condition, *r* = .18 [0.34, 0.02], *p* = 0.24. No relationship was found between the two variables in the Risky ONLY (*r* = −.04 [−0.20, 0.13], *p* = .64) and BOTH Risky/Safe conditions (*r* = .04 [−0.13, 0.2], *p* = .66).

### Discussion

In this study, we replicated and extended Zeelenberg et al. (1996) paper on the consequences of regret aversion in decision making. The findings of the replication were unsuccessful as no support was found for differences in the participants’ strength of risk preference across the three feedback conditions. These results are inconsistent with the original paper which found that participants in Risky ONLY had a higher risk preference than that of participants in Safe ONLY and BOTH Risky/Safe.

This replication is important because Zeelenberg et al. (1996) was one of the first studies to investigate the role of emotional factors such as regret aversion in decision making. Additionally, the original study employed a relatively small sample size (*n* = 76). A power analysis conducted prior to data collection indicated that a sample size of at least 376 was necessary to achieve the power of 95% to test if an effect (*d* = 0.34) of difference in the strength of risk preferences exists in Risky ONLY and BOTH risky/safe conditions. Being a key paper in the literature on regret aversion in decision making, it was crucial to replicate this effect in a well-powered study.

An extension was added to the present replication study to investigate individual differences in participants’ tendency to experience regret across the three feedback conditions. Findings indicated no differences in participants’ tendency to experience regret across the three conditions, and we only found support for a small correlation between the tendency to experience regret and risk preferences of participants in Safe ONLY condition.

## Study 3: Replication of Studies 1a and 2a of Wong and Kwong (2007)

Wong and Kwong (2007) found empirical support for the simultaneous effect of anticipated regret (a prospective factor as it occurs after the decision) and retrospective personal responsibility (a retrospective factor as it occurs before the decision) in driving commitment in escalation situations. The present replication combines studies 1a and 2a of the original paper to adopt a 2 × 2 between-subjects design to study the effects of personal responsibility and regret possibility on escalation of commitment. The original study was conducted on samples of undergraduate students from the University of Hong Kong, and participants were presented with the scenario of an escalation situation in which a target person who was waiting for a bus believed that the bus would come soon, whereas their friend disagreed.

### Methods

This study was preregistered at https://osf.io/24gwu/?view_only=5c0f7cabdb7c443390753b6d78dfead5

#### Participants

A G*Power analysis using the effect sizes of the original study (*d* = 0.81 for personal responsibility and *d* = 0.63 for regret possibility) at 95% power and α = .05 suggested sample sizes of 122 and 262 participants for studies 1a and 2a, respectively (Faul et al., 2007). Using the rule of thumb by Simonsohn (2015), the replication study required at least two and a half times more participants than that of the original study, which is 515 participants (262 [Study 2a] × 2.5 = 515). To account for exclusion and non-completion rates, we recruited 595 U.S. American participants from Amazon MTurk in March 2019. Twenty-seven participants were excluded from analysis as they failed to meet exclusion criteria (see SOM at p. 27), which left a final sample of 568 participants *(M*_age_ = 39.01, *SD*_age_ = 12.25, 285 females, 283 males).

#### Design and Procedure

The replication study has two major deviations from the original study. First, in terms of experimental design, it combines studies 1a and 2a of the original study to form a 2 × 2 between-subjects design. Second, the replication used scenarios and conditions of Study 2a to test the main effects of personal responsibility and regret possibility. Additionally, to improve the clarity of specific dependent variable items, amendments were made to the wording of measures of anticipated regret about withdrawal and persistence. Table 8 includes a summary of the experimental design as well as the dependent variables.

**Table 8.** Summary Table of the Experimental Design (2 × 2 Between-Subject Design) for Wong and Kwong (2007) Replication.

Individual differences Predictor: 13-item subscale of Neuroticism from the Adjective Check List of the Big Five Inventory (Gough & Heilbrun, 1983; McCrae & Costa, 1987)		
	No personal responsibility: Another person was responsible for the prior decision	*Personal responsibility*: Participant was personally responsible for the initiation of the prior decision
No regret possibility: Participants would not know the results of choosing to persist *if they chose to withdraw*.	Condition 1 Title: No personal responsibility—No regret possibility condition	Condition 3 Title: Personal responsibility—No regret possibility condition
Regret possibility: Participants would always know the results from choosing to persist (regardless of choice).	Condition 2 Title: No personal responsibility—Regret possibility condition	Condition 4 Title: Personal responsibility—Regret possibility condition
Dependent variable(s)	*Dependent variable 1*: Anticipated regret about persistence Specific DV item: Estimate the level of regret you will experience if you continued the bet but then the bus failed to arrive within the time limit. [on a scale of 0 (no regret) to 10 (very strong regret)] *Dependent variable 2*: Anticipated regret about withdrawal Specific DV item: [For no regret possibility conditions] Estimate the level of regret you will experience *if you rejected the bet and never knew the outcome* (on a scale of 0 [*no regret*] to 10 [*very strong regret*]) (For regret possibility conditions) Estimate the level of regret you will experience *if you rejected the bet but then the bus arrived within the time limit.* (on a scale of 0 [*no regret*] to 10 [*very strong regret*]) *Dependent variable 3*: Index of escalation tendency Specific DV item: Indicate your willingness to continue the bet by giving a probability rating ranging between 0 (*absolutely no*) and 100 (*absolutely yes*).	

After obtaining informed consent, participants were tested for individual differences in neuroticism. Participants were then randomly assigned to one of the four experimental conditions. In all the conditions, the participants were presented scenarios with escalation situations in which they were asked to imagine that they were waiting for the bus with two friends, Peter and Ken. In the scenario, the participant was asked to imagine that they believed that the bus would arrive soon, and while Ken agreed, Peter disagreed. As a result, Peter and Ken bet $10 on whether the bus would arrive soon (for the scenario refer to Supplemental Material at pp. 10–26). To manipulate personal responsibility, participants were either told that they were personally responsible for the initial bet (in the personal responsibility condition) or that another person (Peter or Ken) was responsible for the initial bet (in the no personal responsibility condition), which was then passed on to the participant. To manipulate regret possibility, participants were told that they would either know the results from choosing to persist after they chose to withdraw from the bet or not.

After reading the scenario, participants answered three questions which were presented in a randomized order. The first question required participants to estimate the level of regret they would experience if they continued the bet, but then the bus failed to arrive within the time limit. For the second question, participants in the *regret possibility* and *no regret possibility* conditions responded to different questions based on whether or not they got feedback on the outcome. Participants in *conditions with no regret possibility* were asked to estimate the level of regret they would experience if they rejected the bet and never knew the outcome. However, for participants in the *conditions with regret possibility*, the second question required them to estimate the level of regret they would experience if they rejected the bet but then the bus arrived within the time limit. The last question asked the participants to indicate their willingness to continue the bet by giving a probability rating ranging between 0 (*absolutely not*) and 100 (*absolutely yes*).

#### Measures

Two items were used to assess the level of regret participants would experience if they “continued the bet but then the bus failed to arrive within the time limit” (anticipated regret about persistence), “rejected the bet and never knew the outcome” (anticipated regret about withdrawal for no regret possibility condition) and “rejected the bet but then the bus arrived within the time limit” (anticipated regret about withdrawal for regret possibility condition) both responded on a 10-point Likert scale (0 = *no regret* to 10 = *very strong regret*; adapted from Courvoisier et al., 2011; Shani & Zeelenberg, 2007). The net anticipated regret about withdrawal was calculated using the anticipated regret about withdrawal (0–10) minus the anticipated regret about persistence (0–10), as in the original study. Escalation of commitment was captured using a 1-item measure asking participants to “indicate their willingness to continue the bet” on a probability rating ranging from 0 = *absolutely not* to 100 = *absolutely yes*. Neuroticism (extension) was measured on a 13-item subscale responded on a 7-point Likert scale (anchored at 1 = *strongly disagree* and 7 = *strongly agree*; McCrae & Costa, 1987), which we then averaged as it had high reliability (Cronbach’s α = .94).

### Results

Based on the original study, higher escalation of commitment was expected in participants in the personal responsibility—regret possibility condition. A two-way ANOVA indicated no support for differences in escalation tendencies across the four conditions, *F*(1,564) = 0.019, *p* = .89, η_p_^2^ = 0.000, 90% CI [0, 0.003]. Additionally, the ANOVA found no support for the effect of personal responsibility, *F*(1,564) = 0.74, *p* = .39, η_p_^2^ = 0.001, [0, 0.011] and regret possibility, *F*(1,564) = 0.36, *p* = .55, η_p_^2^ = 0.001, [0, 0.009] on participant’s escalation tendency.

Next, the relationship between net anticipated regret about withdrawal and the escalation tendency of participants in conditions with regret possibility was examined (refer to Table 9). An independent sample *t*-test indicated no support for differences in net anticipated regret about withdrawal between participants in the two regret possibility conditions, *t*(282) = 0.94, *p* = .35, *d* = 0.11, 95% CI [−0.12, 0.35]. This implies that there exist no differences in the no personal responsibility-regret possibility condition (*M* = −1.39, *SD* = 3.94) and the personal responsibility-regret possibility condition (*M* = −1.83, *SD* = 3.86).

**Table 9.** Results of Independent Sample *t*-Tests on Main Effects of Escalation of Commitment in the Two Regret Possibility Conditions.

Independent sample *t*-tests	*t*	*df*	*p*-value	*d* with 95% CI
Net Anticipated regret about withdrawal	0.91	295	.366	0.11 [−0.12, 0.35]
Anticipated regret about withdrawal	−2.08	282	<.05	−0.25 [−0.48, −0.01]
Anticipated regret about persistence	−3.32	282	<.001	−0.39 [−0.63, −0.16]

An independent samples *t*-test indicated support for differences in anticipated regret about withdrawal between participants in the two regret possibility conditions, *t*(282) = −2.08, *p* < .05, *d* = −0.25, 95% CI [−0.48, −0.01]. This indicates that participants in the personal responsibility—regret possibility condition (*M* = 5.50, *SD* = 3.14) have higher anticipated regret about withdrawal compared to the participants in the no personal responsibility—regret possibility condition (*M* *=* 4.70, *SD* = 3.27). Additionally, support for differences was found in anticipated regret about persistence in the same conditions, *t*(282) = −3.32, *p* < .001, *d* = −0.39, [−0.63, −0.16], with participants in the personal responsibility—regret possibility condition (*M* = 7.32, *SD* = 2.98) have higher anticipated regret about persistence compared to the participants in no personal responsibility—regret possibility condition (*M* *=* 6.10, *SD* = 3.24). These findings are summarized in Table 9.

#### Extension: Individual Differences in Neuroticism

We found no support for an association between neuroticism and escalation of commitment, Pearson’s *r* = −.04, *p* = .376, 95% CI [−0.12, 0.05], and neuroticism and net anticipated regret about withdrawal, *r* = .04, *p* = .317, [−0.04, 0.12]. On the other hand, we found support for a weak positive correlation between neuroticism and anticipated regret about withdrawal, *r* = .19, *p* < .001, [0.11, 0.27]. Additionally, a small positive correlation was also found between neuroticism and anticipated regret about persistence, *r* = .14, *p* = .001, [0.06, 0.22].

### Discussion

This study replicated and extended Wong and Kwong’s (2007) studies 1a and 2a, which examined the role of anticipated regret in decision making under escalation situations. The findings of our replication are inconsistent with the original article as no support was found for the effect of personal responsibility, regret possibility, and their interaction on escalation tendency. It is important to know the results of this replication, as there is a lack of research testing the possible interactions between the effects of personal responsibility and anticipated regret on the escalation of commitment. It is further important to note that there are no known close or very close replications of Wong and Kwong’s (2007) original study.

In their influential article, Wong and Kwong (2007) explored the under-researched (at the time) role of prospective factors in influencing the escalation of commitment. They further suggested that anticipated regret is relevant to all decision makers regardless of whether individuals were personally responsible for the initial decision or not, and thus, the influence of anticipated regret on escalation of commitment is independent as well as higher than that of personal responsibility. Thus, the unsuccessful replication suggests that further investigation is required to examine the interaction of personal responsibility and anticipated regret.

The extension of the present replication study examined the impact of individual differences in neuroticism on the escalation of commitment. In line with existing research (Moon et al., 2003), we found no support for the notion that neuroticism affects escalation of commitment. However, this finding is inconsistent with some previous research which found higher levels of neuroticism to be linked to a lower escalation tendency (Wong & Kwong, 2006). Moreover, we found support for a small positive correlation of neuroticism with anticipated regret about withdrawal and anticipated regret about persistence, indicating higher anticipated regret in participants with high neuroticism levels. Existing literature indicates mixed findings on the relationship between neuroticism and anticipated regret, with a small positive correlation found for consumer purchase regret (Zulkarnain et al., 2018) and a small negative correlation found for Facebook usage (Moore & McElroy, 2012). Overall, in light of these mixed findings, further investigation is necessary to examine the influence of neuroticism on the escalation of commitment.

## General Discussion

The present study is a replication and extension of three influential studies about the escalation of commitment and its psychological drivers.

### Replication Findings

**Figure 1.**
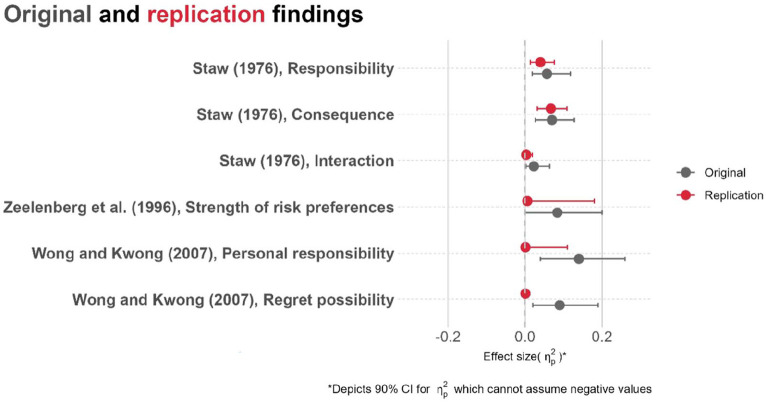
Comparison of original and replicated findings. *Note.* Dots represent effect size point estimates, and lines represent 90% confidence intervals around η_p_^2^ (which cannot assume negative values).

Table 10 and Figure 1 provide a quantitative comparison between the original and replication studies. We found partial support for Staw’s (1976) study, which found that high personal responsibility and negative consequences lead to higher escalation to commitment. In our replication, we found support for both personal responsibility and consequences on escalation to commitment, but the effect of consequences was found in the opposite direction. For the replication of Zeelenberg et al. (1996) in study 2, no support was found for the consequences of regret aversion on decision making. Lastly, for study 3, we failed to replicate Wong and Kwong’s (2007) study as no support was found for the effect of personal responsibility, regret possibility, and their interaction on escalation tendency.

**Table 10.** Interpretation of Replication Findings (Based on LeBel et al. (2019)).

Original paper	Study	Original effect size	Original 95% CI for ES^a^	Replicated effect size	Replicated 95% CI for ES^a^	Interpretation
Wong and Kwong (2007)	Personal responsibility	η_p_^2^ = 0.14	[0.04, 0.26]	η_p_^2^ = 0.001	[0, 0.11]	No signal, inconsistent
	Regret possibility	η_p_^2^ = 0.09	[0.02, 0.19]	η_p_^2^ = 0.001	[0, 0.009]	No signal, inconsistent
Zeelenberg et al. (1996)	Strength of risk preferences	η_p_^2^ = 0.084	[0, 0.20]	η_p_^2^ = 0.006	[0, 0.18]	No signal, consistent
Staw (1976)	Responsibility	η_p_^2^ = 0.057	[0.019, 0.118]	η_p_^2^ = 0.040	[0.014, 0.076]	Signal, consistent
	Consequence	η_p_^2^ = 0.070	[0.027, 0.128]	η_p_^2^ = 0.067	[0.032, 0.109]	Signal, inconsistent^3^
	Interaction	η_p_^2^ = 0.023	[0.002, 0.064]	η_p_^2^ = 0.003	[0, 0.019]	No signal, inconsistent

a We used 90% CIs for η_p_^2^ since it cannot assume negative values.

The present study further added extensions to examine the influence of individual differences in neuroticism on the escalation of commitment (study 3) and the tendency to experience regret on decision making (study 2). No support for individual differences in neuroticism was found in study 3, whereas study 2 found support for a small correlation between the tendency to experience regret and risk seeking for participants in the Safe ONLY condition.

These studies have been influential in the field of escalation of commitment and decision making, as is evidenced by the number of citations and provide a comprehensive framework examining the cognitive (i.e., Staw, 1976), emotional (i.e., Zeelenberg et al., 1996), and motivational (i.e., Wong & Kwong, 2007) factors that drive escalated commitment to a prior decision. Thus, failing to replicate these findings raises questions regarding the robustness of the existing theoretical explanations to understand the escalation of commitment and implies that further research is required. In this replication, we found support for self-justification in driving escalation of commitment through higher commitment to personally responsible prior decisions with positive consequences (Staw, 1976). On the other hand, no support was found for anticipated regret (Wong & Kwong, 2007) and regret aversion (Zeelenberg et al., 1996) as drivers of escalated commitment. This suggests that motivational factors (i.e., anticipated regret) might not always drive escalation and emotional factors (i.e., regret aversion) might not be universal decision-making biases.

Evidence along these lines is reflected in existing literature, which suggests mixed results on the effects of personal responsibility (Davis & Bobko, 1986; Singer & Singer, 1985, 2001) and regret possibility (Heath, 1995; Karlsson et al., 2005; Parks & Conlon, 1990) in escalation situations. These studies suggest that personal responsibility may not always lead to escalation of commitment, and the role of mediators such as variations in contexts and locus of control has been examined (Davis & Bobko, 1986; Singer & Singer, 1985). This is consistent with our findings, as we found mixed effects of personal responsibility in the replication of Staw (1976) and Wong and Kwong (2007). Additionally, studies have found that regret possibility may lead to de-escalation (Heath, 1995; Parks & Conlon, 1990), while in line with our findings from the replication of Staw (1976), others have reported an opposite effect (Karlsson et al., 2005). For the role of emotional factors, research suggests other emotions such as fear of failure (Tetlock, 1985) and hope for recovery (Sharot, 2011) might be stronger drivers of persistent risky behavior compared to regret aversion as suggested by Zeelenberg et al. (1996). Overall, further investigation is required to understand this phenomenon.

### Inconsistent Findings: Discussion of Possible Factors

In this section, we discuss factors that might help us explain the discrepancy of findings between the original studies and the replications.

#### Participant Demographics and Cultural Differences

One reason for the discrepancy in findings may be accounted to the differences in participant demographics in the original and replication studies. For instance, in the replication of Wong and Kwong (2007) participants were recruited online through MTurk from the United States from diverse backgrounds and age groups, whereas participants in the original study were undergraduate students from Hong Kong. Previous research has shown that the effects of anticipated regret, escalation of commitment and neuroticism were applicable to people of different countries, and thus changes in participant demographics should not lead to large differences in results (Sleesman et al., 2012; Wong & Kwong, 2007; Zeelenberg et al., 1996). However, researchers have also found cultural differences in escalation behavior (Keil et al., 2000; Liang et al., 2014). In addition, a study by Wong (2005) found that the age of participants influences decision making in escalation situations. Our study 3 is restricted in terms of testing if differences in findings can be accounted age and cultural differences of participants, and therefore we suggest that future replications of Wong and Kwong (2007) recruit participants similar to those of the original study.

Additionally, unlike the original study, participants in the replication study were native English speakers and thus minor amendments were made to improve the clarity of the scenarios for American participants. Despite being minor, the differences in the information presented to the participants might have affected our findings.

Difference in participant demographics might also explain the inconsistent findings for the replication of Zeelenberg at al. (1996) and Staw’s (1976) study. Participants for study 1 and study 2 were U.S. American participants recruited online through MTurk, whereas the original authors recruited participants from the University of Illinois and the University of Amsterdam for study 1 and study 2, respectively.

Lastly, it is worth noting that using differences in participant demographics to explain failed replications reduces the generalizability of the original findings. This is because the importance of a finding in understanding human behavior is reduced if it is found to be limited to a certain population and cannot be replicated in diverse samples. This further undermines the broader contribution of these findings (Schimmelpfennig et al., 2024).

#### Time

The discrepancy of findings in the original studies and the present replication might be attributed to the passage of time and different samples. This time-lag between the original study and the present replications might be used as an explanation of inconsistent findings for Zeelenberg et al. (1996) and Staw (1976). However, this argument is weaker for Wong and Kwong (2007), which is more recent. Additionally, other studies in the field of judgment and decision making have been successfully replicated despite a similar time-lag (Zhu & Feldman, 2023; Ziano, Xiao, et al., 2021).

#### Procedures

Another reason for the inconsistent findings might be accounted to different procedures adopted in the original studies and the present replications. For instance, one major difference in study 3 is that it combined studies 1a and 2a of Wong and Kwong (2007) to adopt a 2 × 2 between-subjects design to examine the effects of personal responsibility and regret possibility on escalation of commitment. This allowed us to further explore the interaction of these terms on the escalation of commitment. The original study, on the other hand, studied the main effects of personal responsibility and anticipated regret in two separate studies with different scenarios and dependent variables. The present replication adopted scenarios from study 2a (see Supplemental Material at p. 10 for the scenarios). The scenarios used in studies 1a and 2a of the original paper had different parameters in terms of the betting amount and betting duration. Since identical parameters are needed for an effective comparison between the scenarios, this was a necessary modification. These modifications that allowed us to replicate the effects of the original study with a higher power are not sufficient to explain the discrepancy in findings.

Another broader procedural difference between the original studies and the replications is that the replications were conducted online on samples recruited through MTurk. On the contrary, the original studies were conducted in university campuses and on a sample of university students using the paper-pencil method of data collection. This difference, however, is not enough to explain the discrepancy in findings, as several prior studies have successfully replicated findings originally obtained with U.S. American students with online participants on platforms like MTurk and Prolific (Ziano, Kong, et al., 2020; Ziano, Li, et al., 2021; Ziano, Mok, et al., 2020). Moreover, accounting inconsistency in findings to these procedural differences limits the generalizability of the findings and further undermines their contribution to literature.

### Directions for Future Research

The findings from our replication raise questions regarding the robustness of the cognitive, emotional, and motivational factors in providing a comprehensive framework to understand escalation of commitment. Failed replication implies that escalation of commitment might be a more complex phenomenon than theorized, and future investigations are necessary to examine other variables to understand this phenomenon. We suggest that future researchers adopt a cross-cultural replication approach to determine whether escalated behavior and regret effects vary by societal norms. For instance, in hierarchical cultures with high power distance (Hofstede, 2001), escalated behavior might be driven by social pressure rather than self-justification. Additionally, to improve the external validity of these effects, researchers can further aim to replicate existing research that adopts more realistic decision environments. This includes, for example, Zeelenberg and Pieters’ (2004) work on real-life lottery participation decisions. Additionally, inconsistent findings such as no support for the effects of anticipated regret and regret aversion in our replication might be accounted to several other factors such as participant demographics and cultural differences, which we have discussed in the above section.

## Conclusion

Overall, the findings from our replication challenge the robustness of the existing theoretical explanations to understand escalation of commitment, as we failed to replicate key findings from Staw (1976), Zeelenberg et al. (1996), and Wong and Kwong (2007). While we found support for the role of self-justification in driving escalation of commitment (Staw, 1976), no support was found for regret aversion (Zeelenberg et al., 1996) or anticipated regret (Wong & Kwong, 2007) as drivers of escalated commitment to a prior decision. These findings suggest that escalation of commitment cannot be completely explained through cognitive, emotional, and motivational factors as theorized, and other factors must be examined to have a better understanding of the phenomenon. We suggest that future research take a context-dependent approach and use more realistic decision environments to understand escalation of commitment.

Importantly, we highlight the importance of replication studies in driving psychological science as they help build confidence in reliable effects and test whether effects hold over time and across contexts. To this end, we encourage future replication attempts of these effects and other classic studies.

## Supplemental Material

sj-docx-1-psp-10.1177_01461672251345021 – Supplemental material for A Replication and Extension of Three Studies Investigating Escalation of Commitment and Regret Aversion Supplemental material, sj-docx-1-psp-10.1177_01461672251345021 for A Replication and Extension of Three Studies Investigating Escalation of Commitment and Regret Aversion by Nandita Dhanda, Ignazio Ziano, Kam Hoi Ching, Angela Kwun Lan Phuong, Yuen Ting Lo, Man Kin Yip, Bill Jiaxin Shi and Boley Cheng in Personality and Social Psychology Bulletin

## Notes

1. Escalation of commitment is sometimes also referred to as “sunk cost bias” or “sunk cost effect.”

2. Reported this way in the original article.

3. For Staw (1976), the effect is classified as inconsistent even though the 95% CI for the replication includes the effect size from the original paper. This is because the appropriate effect size for the consequence variable is η_p_^2^, which can only take on positive values whereas the Lebel et al. (2019) classification is based on using Pearson’s correlation coefficient *r* as the effect size which can take both negative and positive values.
