# Supplementary material for: A Replication and Extension of Three Studies Investigating Escalation of Commitment and Regret Aversion
Source: Pers Soc Psychol Bull. 2025 Jun 19;52(9):2677–92. doi: 10.1177/01461672251345021 (PMC13392164; doi:10.1177/01461672251345021)
Supplement: sj-docx-1-psp-10.1177_01461672251345021 – Supplemental material for A Replication and Extension of Three Studies Investigating Escalation of Commitment and Regret Aversion [file sj-docx-1-psp-10.1177_01461672251345021.docx]

**A Replication and Extension of Three Studies Investigating Escalation of Commitment and Regret Aversion**

**Supplementary Online Materials**

**Table of contents**

[**Study 1: Staw (1976)** 2](#_Toc197300402)

[**Deviations from pre-registration** 2](#_Toc197300403)

[**Participant demographic.** 2](#_Toc197300404)

[**Scenarios** 2](#_Toc197300405)

[**Other factor checks** 7](#_Toc197300406)

[**Study 2: Zeelenberg et al. (1996)** 8](#_Toc197300407)

[**Deviations from pre-registration.** 8](#_Toc197300408)

[**Exclusion criteria.** 8](#_Toc197300409)

[**Reasons for choice** 8](#_Toc197300410)

[**Study 3: Wong and Kwong (2007)** 10](#_Toc197300411)

[**Deviations from pre-registration.** 10](#_Toc197300412)

[**Materials used in the experiments.** 10](#_Toc197300413)

[**Participant exclusion.** 26](#_Toc197300414)

[**Main effects of personal responsibility and regret possibility** 27](#_Toc197300415)

[**Replication exploratory analysis** 28](#_Toc197300416)

[**Extensions** 30](#_Toc197300417)

**Study 1: Staw (1976)**

**Deviations from pre-registration.** No deviations.

**Participant demographic.** Most participants were middle class (179) and working class (87), followed by upper middle class (30) and lower middle class (80), and only a few in lower class (22) and upper class (2).

**Scenarios:** High personal responsibility, consumer up, industrial down

The A&S company is a large technologically-oriented firm. The profitability of the company has started to decline over several preceding years. The directors of the company have agreed that one of the major reasons for the decline of corporate earnings lay in aspect of the firm's program of research and development (R&D). To improve the corporate earnings of the company, the directors have concluded that 10 million dollars of additional R&D funds would be made available to its major operating divisions.

 The extra investment of 10 million dollars should be only allocated to one of the two majors corporate divisions: Consumer Products Industrial Products
 
Now, as the Financial Vice President of the A&S company, you are asked to make the allocation choice, to see which of the two corporate divisions should receive the R&D funds, on the basis of the potential benefit the R&D fundings will have on the future earnings of the division. Financial history (i.e. sales and earnings data of ten prior years) of the two corporate divisions was illustrated in the two tables below, respectively.

 Please choose and explain your decision...

s1976-hr-cu-int2
The following is the report you received about your **consumer products division**:


(sales and earnings are in millions, earnings marked in parentheses indicate losses)


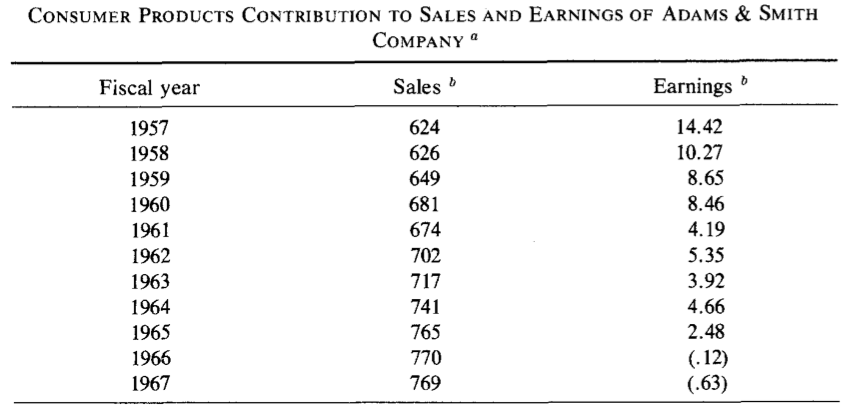


s1976-hr-cu-int3
The following is the report you received about your **industrial products division**:

(sales and earnings are in millions, earnings marked in parentheses indicate losses)


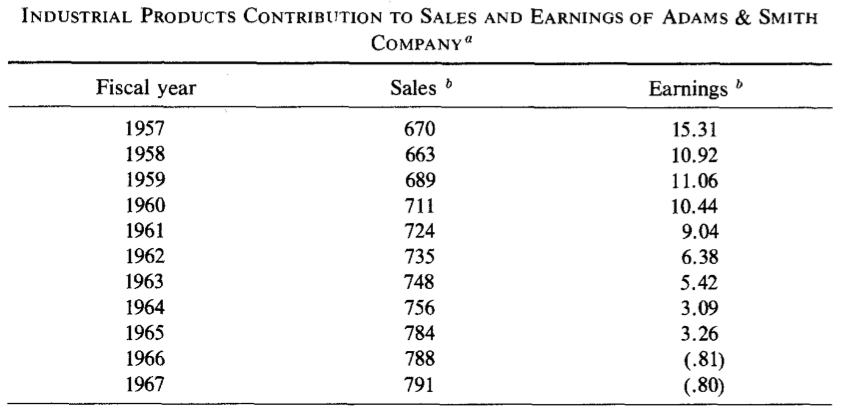


| 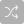 |
| --- |

s1976-hr-cu-choice1 After analysing the financial information of the two corporate divisions (Consumer Products and Industrial Products), please make a decision which division to allocate the 10 million dollars of R&D funds to...

- Consumer Products (1)
- Industrial Products (2)

| Page Break |  |
| --- | --- |

s1976-hr-cu-c1-expla
You selected allocating 10 million dollars to **${s1976-hr-cu-choice1/ChoiceGroup/SelectedChoices} division**
 
Please explain your allocation decision (very briefly, 1-2 sentences)

________________________________________________________________

| Page Break |  |
| --- | --- |

s1976-hr-cu-int4
Five years after the decision of the allocation of R&D funds you have made, the program is up for re-evaluation.


Financial situation (i.e. sales and earnings) of the two divisions throughout the five years is given below.

s1976-hr-cu-int5


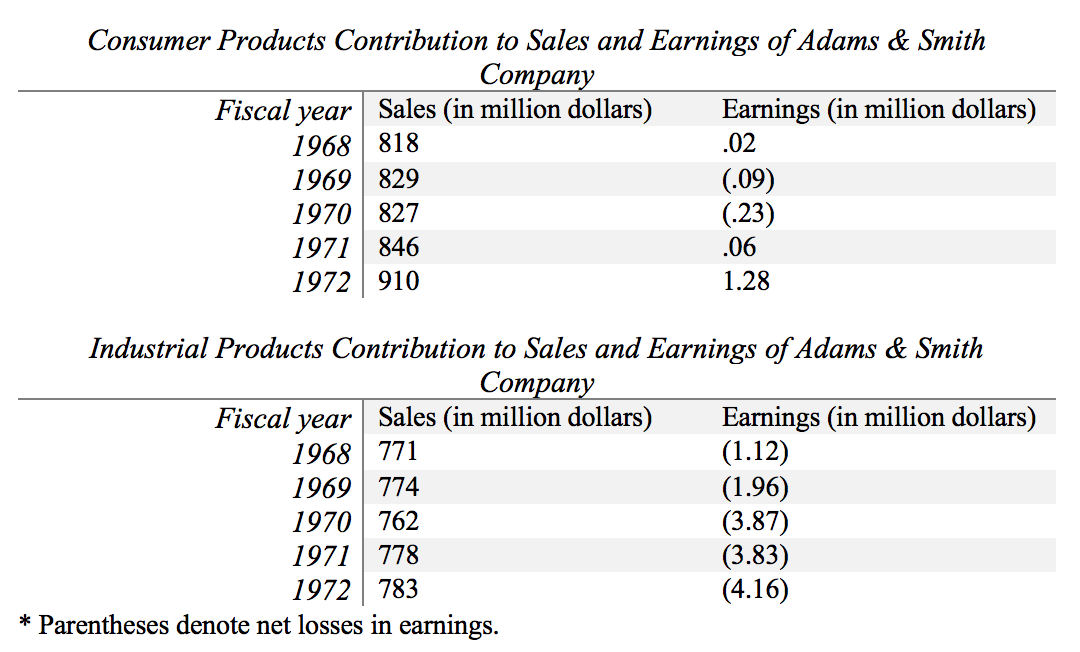


| 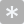 |
| --- |

s1976-hr-cu-allocate
**In your previous decision, you allocated 10 million dollars to ${s1976-hr-cu-choice1/ChoiceGroup/SelectedChoices} division.**
 
The directors believe that there is a greater need for expenditure on research and development, hence, an amount of 20 million dollars will be made available for extra R&D funds.
 
**You, as the Financial Vice President, are again required to make a proper decision on allocating the 20 million dollars among the two corporate divisions, on the basis of future contribution to the company.**
 
This time, you can divide the funds in any way you wish among the two divisions.
 Make sure the total amount of money allocated in the two divisions is equal to 20 million dollars.
 
Please choose and explain your decision...

Amount of funds (in million dollars) allocating to **Consumer Products** : _______ (1)

Amount of funds (in million dollars) allocating to **Industrial Products** : _______ (2)

Total : ________

s1976-hr-cu-c2-expla Please explain your allocation decision (very briefly, 1-2 sentences)

________________________________________________________________

### **Other factor checks**

To determine if there were any differences to commitment based on First Choice, an independent samples t-test was used. This was done in order to check whether the choices themselves were considered equal, or if one was significantly preferred over the other, thus affecting the results. Results indicated no significant differences in commitment to the prior financial decision based on first choice (*t*(398) = 0.28, p = .78, *d* = 0.03 [-0.17, 0.23]). This indicates that no significant differences exists whether the money was allocated to the consumer division (M = 9.07, SD = 5.10) or industrial division (M = 8.93, SD = 5.04) at the first financial decision D1.

Next, an independent samples t-test was conducted to determine if any differences exist in Financial Data provided to the provided as a variable. This variable includes two levels, Consumer > Industrial (c > i) and Industrial > Consumer (i > c). Results indicated no significant differences in commitment to the prior financial decision based on financial data (*t*(398) = -0.55, p = .58, *d* = -0.06 [-0.25, 0.14]). This indicates that no significant differences exists whether fund allocation was c > i (M = 8.85, SD = 5.12) or i > c (M = 9.13, SD = 5.00) at the first financial decision D1.

# **Study 2: Zeelenberg et al. (1996)**

**Deviations from pre-registration.** No deviations.

### **Exclusion criteria.**

1. Participants indicating a low proficiency of English (self-report<5, on a 1-7 scale)
2. Participants who self-report not being serious about filling in the survey (self-report<4, on a 1-5 scale).
3. Participants who correctly guessed the hypothesis of this study in the funnelling section.
4. Participants who failed to complete the survey.

### **Reasons for choice**

The study also involved asking participant to write down a reason for their choice. These written justifications provide further support for the participants’ low risk preference in decision making. Due to limited resources, our study was unable to use inter-rater reliability to categorise the justifications. Upon initial review, we found that the justifications made by the participants could be sorted into eight categories. The categories are as follows with examples:

1. Regret aversion (mentioned feedback and a desire to avoid regret)
2. Risk aversion (prefers the choice with a higher probability of winning and mentioned a desire to avoid regret)
3. Risk seeking (prefers to take risks and would risk to gain more from the gambles)
4. High value set for X (participants who placed a value for X that was much higher than that of the money received for the original gamble, and stated that to be the reason of their choice)
5. Scenario lacked generalisability (participants who decided to take a risk or randomly choose options because the gamble is not real, no actual loss or gain involved)
6. Lack clarity (participants who were unclear in their justifications)
7. Want to compare gambles (participants who actively want to compare the outcomes of both gambles)
8. Others (any other reason, e.g. personal preference)

Overall, the most common justification provided by participants in the three conditions was (2) Risk aversion (69.4%). These participants believed that the choice with the higher probability for gain is the better choice and they preferred not to take risks in gambling. For example, a participant in Risky ONLY who chose Gamble S wrote that, “65% chance of winning something, even a lesser amount, is better than 65% chance of winning nothing”. Upon reviewing the risk preference of participants, this justification is a likely reason for the large number of participants choosing Gamble S over Gamble R, as in all three conditions, most participants used this justification ([Risky ONLY] = 65.5%; [Safe ONLY] = 69.1%; [BOTH Risky/Safe] = 73.3%). Contrary to the results of the original study, only 2.5% of the participants reported regret aversion to be the reason for their chosen gamble.

Table 1 shows the counts and percentages for each justification across the three feedback conditions.

Table 1

Frequency table of categorised justifications for their choice made by participants

| Conditions  With (%)1 | Regret aversion | Risk aversion | Risk seeking | High value set for X | Scenario lacked generalisability | Lacked clarity | Wanted to compare gambles | Others | Total |
| --- | --- | --- | --- | --- | --- | --- | --- | --- | --- |
| Risky ONLY | 4 (2.8%) | 95 (65.5%) | 14 (9.7%) | 5 (3.4%) | 2 (1.4%) | 10 (6.9%) | 8 (5.5%) | 7 (4.8%) | 145 |
| Safe ONLY | 7 (4.7%) | 103 (69.1%) | 15  (10.1%) | 3 (2.0%) | 0 (0.0%) | 10 (6.7%) | 6 (4.0%) | 5 (3.4%) | 149 |
| BOTH Risky/ Safe | 0 (0.0%) | 110 (73.3%) | 20 (13.3%) | 1 (0.7%) | 2 (1.3%) | 8 (5.3%) | 0 (0.0%) | 9 (6.0%) | 150 |
| Total | 11 (2.5%) | 308  (69.4%) | 49 (11.0%) | 9  (2.0%) | 4 (0.9%) | 28 (6.3%) | 14 (3.2%) | 21 (4.7%) | 444 |

^1^ Percentages in italics *(%)*: percentage of choice frequency within conditions

## **Study 3: Wong and Kwong (2007)**

**Deviations from pre-registration.** No deviations from the pre-registration.

### **Materials used in the experiments.**

This section includes the materials shown to participants in the survey.

*Italic* words in parentheses are remarks to the readers of this document and were not shown to participants.

Differences in scenarios between the conditions are bolded and underlined.

#### Introduction

*Instructions*

This study has 2 parts.

The first part about your personality contains 28 questions with multiple choice. The second part contains 11 questions about a decision-making scenario.
Please read the scenario items carefully. There are no right or wrong answers, answer to the best of your understanding and according to your own preferences.

#### Exploratory study – Neuroticism subscale of Adjective Check List

*Instructions*

Please read the descriptions carefully.

On a scale of 1 (completely disagree) to 7 (completely agree), please rate the following adjectives that could best describe you.

Reminder: There are no right or wrong answers, answer to the best of your understanding and according to your own preferences.
*(These items are presented in a randomised order)*

Q1. Calm; Q2. Worrying; Q3. At ease; Q4. Nervous; Q5. Relaxed; Q6. High strung; Q7. Unemotional; Q8. Emotional; Q9. Even-tempered; Q10. Temperamental; Q11. Secure; Q12. Insecure; Q13. Self-satisfied; Q14. Self-pitying; Q15. Patient; Q16. Impatient; Q17. Not envious; Q18. Envious/ jealous; Q19. Comfortable; Q20. Self-conscious; Q21. Not impulse ridden; Q22. Impulse ridden; Q23. Hardy; Q24. Vulnerable; Q25. Objective; Q26. Subjective.

Q27. Physically alive; Q28. Physically dead *(These two items are attention checks)*

#### Condition 1: No personal responsibility-No regret possibility

Comprehension check

*(Participants need to provide a correct answer to proceed)*

*Instructions*

Please read the description carefully and answer the questions that follow.

One day, you and two friends, Peter and Ken were on the way to take a bus together. Unfortunately, by the time you reached the bus stop, the bus you wanted to take had already left. You needed to wait for the next bus. You remembered that the bus service was quite frequent and said, “This is a frequent bus. We won’t have to wait for too long. The next bus should arrive in 10 minutes.” Although Ken agreed with you, Peter expressed doubt. Peter said, “I don’t think so. I remember that the service of this bus is actually quite infrequent. Last time, I waited for around 40 minutes for the next bus.” Ken and Peter then engaged in a debate about the frequency of the bus.

 Consequently, Ken and Peter decided to bet on it. If the bus arrived within the next 15 minutes, Peter would pay $10 to Ken. However, if the bus failed to arrive within the next 15 minutes, Ken would pay $10 to Peter.

After 15 minutes of waiting, the bus still had not arrived. Ken lost $10 to Peter.

 Then, Peter suggested another bet. Ken passed the offer on to you. 
 **You could reject the bet and leave the bus stop to take the train instead. As a result, you would not know if the bus arrived within the remaining 15 minutes.**
 The choice: If you accepted Peter's suggestion and proceeded with the bet, and the bus arrived within the next 15 minutes, Peter would give you the $10 he had gained. However, if the bus still failed to arrive within the next 15 minutes, you would pay Peter $10. If you rejected Peter's suggestion and the bet, you would not risk losing an additional $10 to Peter if the bus failed to arrive. However, you would lose any chance of gaining $10 from Peter. *(Order of answers were randomised)*

Q1. Who decided to have the first bet?

- Peter and you
- Ken and you
- Peter and Ken *(correct answer)*

Q2. In the first bet, how much would Peter pay if the bus had arrived in the first 15 minutes?

- $10 *(correct answer)*
- $15

Q3. In the first bet, how much would Peter be paid if the bus did not arrive in the first 15 minutes?

- $10 *(correct answer)*
- $15

Q4. Did the bus arrive before Peter proposed the second bet?

- Yes
- No *(correct answer)*

Q5. Who would be participating in the proposed second bet?

- Peter and you *(correct answer)*
- Ken and you
- Peter and Ken

Q6. In the second bet, how much would Peter pay if the bus arrived within the time limit?

- $5
- $10 *(correct answer)*
- $15

Q7. In the second bet, how much would Peter be paid if the bus did not arrive within the time limit?

- $5
- $10 *(correct answer)*
- $15

Q8. In the second bet, if you declined the bet would you ever know if the bus arrived within the time limit?

- Yes
- No *(correct answer)*

Main questions

*(Instructions)*

You will now be asked to answer 3 questions based on the scenario you just read.

Following is a reminder of the scenario: *(same scenario as above in comprehension check)*

Q1. Estimate the level of regret you will experience **if you ...** Continued the bet but then the bus failed to arrive within the time limit

- 0 (no regret)
- 1
- 2
- 3
- 4
- 5
- 6
- 7
- 8
- 9
- 10 (very strong regret)

Q2. Estimate the level of regret you will experience **if you ...** Rejected the bet and never knew the outcome.

- 0 (no regret)
- 1
- 2
- 3
- 4
- 5
- 6
- 7
- 8
- 9
- 10 (very strong regret)

Q3. Indicate your willingness to continue the bet by giving a probability rating ranging between 0 (*absolutely no*) and 100 (*absolutely yes*).
*Reminder: The probability rating should be a number ranging between 0 to 100.*

#### Condition 2: No personal responsibility- Regret possibility

Comprehension check

*(Participants need to provide a correct answer to proceed)*

*Instructions*

Please read the description carefully and answer the questions that follow.

One day, you and two friends, Peter and Ken were on the way to take a bus together. Unfortunately, by the time you reached the bus stop, the bus you wanted to take had already left. You needed to wait for the next bus. You remembered that the bus service was quite frequent and said, “This is a frequent bus. We won’t have to wait for too long. The next bus should arrive in 10 minutes.” Although Ken agreed with you, Peter expressed doubt. Peter said, “I don’t think so. I remember that the service of this bus is actually quite infrequent. Last time, I waited for around 40 minutes for the next bus.” Ken and Peter then engaged in a debate about the frequency of the bus.
  
 Consequently, **Ken and Peter both decided to bet on it.** If the bus arrived within the next 15 minutes, Peter would pay $10 to Ken. However, if the bus failed to arrive within the next 15 minutes, Ken would pay $10 to Peter.
  
 After 15 minutes of waiting, the bus still had not arrived. Ken lost $10 to Peter.

 **Then, Peter suggested another bet. Ken passed the offer on to you. You would wait an additional 15 minutes to see if the next bus arrives.**

The choice: If you accepted Peter's suggestion and proceeded with the bet, and the bus arrived within the next 15 minutes, Peter would give you the $10 he had gained. However, if the bus still failed to arrive within the next 15 minutes, you would pay Peter $10. If you rejected Peter's suggestion and the bet, you would wait for an additional 15 minutes for the next bus, but you would not risk losing an additional $10 to Peter if the bus failed to arrive. However, you would lose any chance of gaining $10 from Peter. *(Order of answers were randomised)*

Q1. Who decided to have the first bet?

- Peter and you
- Ken and you
- Peter and Ken *(correct answer)*

Q2. In the first bet, how much would Peter pay if the bus had arrived in the first 15 minutes?

- $10 *(correct answer)*
- $15

Q3. In the first bet, how much would Peter be paid if the bus did not arrive in the first 15 minutes?

- $10 *(correct answer)*
- $15

Q4. Did the bus arrive before Peter proposed the second bet?

- Yes
- No *(correct answer)*

Q5. Who would be participating in the proposed second bet?

- Peter and you *(correct answer)*
- Ken and you
- Peter and Ken

Q6. In the second bet, how much would Peter pay if the bus arrived within the time limit?

- $5
- $10 *(correct answer)*
- $15

Q7. In the second bet, how much would Peter be paid if the bus did not arrive within the time limit?

- $5
- $10 *(correct answer)*
- $15

Q8. In the second bet, if you declined the bet would you ever know if the bus arrived within the time limit?

- Yes *(correct answer)*
- No

Main questions

*(Instructions)*

You will now be asked to answer 3 questions based on the scenario you just read.

Following is a reminder of the scenario: *(same scenario as above in comprehension check)*

Q1. Estimate the level of regret you will experience **if you ...** Continued the bet but then the bus failed to arrive within the time limit

- 0 (no regret)
- 1
- 2
- 3
- 4
- 5
- 6
- 7
- 8
- 9
- 10 (very strong regret)

Q2. Estimate the level of regret you will experience **if you ...** Rejected the bet and never knew the outcome.

- 0 (no regret)
- 1
- 2
- 3
- 4
- 5
- 6
- 7
- 8
- 9
- 10 (very strong regret)

Q3. Indicate your willingness to continue the bet by giving a probability rating ranging between 0 (*absolutely no*) and 100 (*absolutely yes*).
*Reminder: The probability rating should be a number ranging between 0 to 100.*

#### Condition 3: Personal responsibility-No regret possibility

Comprehension check

*(Participants need to provide a correct answer to proceed)*

*Instructions*

Please read the description carefully and answer the questions that follow.

One day, you and two friends, Peter and Ken were on the way to take a bus together. Unfortunately, by the time you reached the bus stop, the bus you wanted to take had already left. You needed to wait for the next bus. You remembered that the bus service was quite frequent and said, “This is a frequent bus. We won’t have to wait for too long. The next bus should arrive in 10 minutes.” Although Ken agreed with you, Peter expressed doubt. Peter said, “I don’t think so. I remember that the service of this bus is actually quite infrequent. Last time, I waited for around 40 minutes for the next bus.” You and Peter then engaged in a debate about the frequency of the bus.

Consequently, **you and Peter decided to bet on it.** You agreed to wait at the bus stop for 15 minutes and pay $1 to Peter every minute in this 15-minute period until the bus arrived. Thus, if the bus did not arrive within 15 minutes, you would lose $15 in total to Peter. However, if the bus arrived within 15 minutes, he would pay $15 to you but would not return any money that you had given him previously.
  
 After 10 minutes of waiting, the bus still had not arrived and you had already paid $10 to Peter. **Now, Peter suggested that you could stop the bet and leave the bus-stop to take the train instead. As a result, you would not know if the bus arrived within the remaining 5 minutes.**

The choice: If you accepted Peter’s suggestion and terminated the bet, you would not risk losing an additional $5 to Peter if the bus failed to arrive. However, if the bus managed to arrive within the next 5 minutes, Peter would also not pay $15 to you. Alternatively, you could choose to continue the bet and wait 5 minutes longer. In doing so, you would risk losing an additional $5 to Peter if the bus failed to arrive. On the other hand, if the bus managed to arrive within the remaining 5 minutes, you would gain $15 from Peter.  *(Order of answers were randomised)*

Q1. Who decided to have the first bet?

- Peter and you *(correct answer)*
- Ken and you
- Peter and Ken

Q2. In the first bet, how much would Peter pay if the bus had arrived in the first 15 minutes?

- $10 *(correct answer)*
- $15

Q3. In the first bet, how much would Peter be paid if the bus did not arrive in the first 15 minutes?

- $10 *(correct answer)*
- $15

Q4. Did the bus arrive before Peter proposed the second bet?

- Yes
- No *(correct answer)*

Q5. Who would be participating in the proposed second bet?

- Peter and you *(correct answer)*
- Ken and you
- Peter and Ken

Q6. In the second bet, how much would Peter pay if the bus arrived within the time limit?

- $5
- $10 *(correct answer)*
- $15

Q7. In the second bet, how much would Peter be paid if the bus did not arrive within the time limit?

- $5
- $10 *(correct answer)*
- $15

Q8. In the second bet, if you declined the bet would you ever know if the bus arrived within the time limit?

- Yes
- No *(correct answer)*

Main questions

*(Instructions)*

You will now be asked to answer 3 questions based on the scenario you just read.

Following is a reminder of the scenario: *(same scenario as above in comprehension check)*

Q1. Estimate the level of regret you will experience **if you ...** Continued the bet but then the bus failed to arrive within the time limit

- 0 (no regret)
- 1
- 2
- 3
- 4
- 5
- 6
- 7
- 8
- 9
- 10 (very strong regret)

Q2. Estimate the level of regret you will experience **if you ...** Rejected the bet and never knew the outcome.

- 0 (no regret)
- 1
- 2
- 3
- 4
- 5
- 6
- 7
- 8
- 9
- 10 (very strong regret)

Q3. Indicate your willingness to continue the bet by giving a probability rating ranging between 0 (*absolutely no*) and 100 (*absolutely yes*).
*Reminder: The probability rating should be a number ranging between 0 to 100.*

#### Condition 4: Personal responsibility-Regret possibility

Comprehension check

*(Participants need to provide a correct answer to proceed)*

*Instructions*

Please read the description carefully and answer the questions that follow.

One day, you and two friends, Peter and Ken were on the way to take a bus together. Unfortunately, by the time you reached the bus stop, the bus you wanted to take had already left. You needed to wait for the next bus. You remembered that the bus service was quite frequent and said, “This is a frequent bus. We won’t have to wait for too long. The next bus should arrive in 10 minutes.” Although Ken agreed with you, Peter expressed doubt. Peter said, “I don’t think so. I remember that the service of this bus is actually quite infrequent. Last time, I waited for around 40 minutes for the next bus.” You and Peter then engaged in a debate about the frequency of the bus.

Consequently, **you and Peter decided to bet on it.** If the bus arrived within the next 15 minutes, Peter would pay $10 to you. However, if the bus failed to arrive within the next 15 minutes, you would pay $10 to Peter.
  
 After 15 minutes of waiting, the bus still had not arrived. You lost $10 to Peter.

 **Then, Peter suggested another bet. You would wait an additional 15 minutes to see if the next bus arrives.**

The choice: If you accepted Peter's suggestion and proceeded with the bet, and the bus arrived within the next 15 minutes, Peter would give you the $10 he had gained. However, if the bus still failed to arrive within the next 15 minutes, you would pay Peter an additional $10. If you rejected Peter's suggestion and the bet, you would wait for an additional 15 minutes for the next bus, but you would not risk losing an additional $10 to Peter if the bus failed to arrive. However, you would lose any chance of gaining back the $10 from Peter.  *(Order of answers were randomised)*

Q1. Who decided to have the first bet?

- Peter and you *(correct answer)*
- Ken and you
- Peter and Ken

Q2. In the first bet, how much would Peter pay if the bus had arrived in the first 15 minutes?

- $10 *(correct answer)*
- $15

Q3. In the first bet, how much would Peter be paid if the bus did not arrive in the first 15 minutes?

- $10 *(correct answer)*
- $15

Q4. Did the bus arrive before Peter proposed the second bet?

- Yes
- No *(correct answer)*

Q5. Who would be participating in the proposed second bet?

- Peter and you *(correct answer)*
- Ken and you
- Peter and Ken

Q6. In the second bet, how much would Peter pay if the bus arrived within the time limit?

- $5
- $10 *(correct answer)*
- $15

Q7. In the second bet, how much would Peter be paid if the bus did not arrive within the time limit?

- $5
- $10 *(correct answer)*
- $15

Q8. In the second bet, if you declined the bet would you ever know if the bus arrived within the time limit?

- Yes *(correct answer)*
- No

Main questions

*(Instructions)*

You will now be asked to answer 3 questions based on the scenario you just read.

Following is a reminder of the scenario: *(same scenario as above in comprehension check)*

Q1. Estimate the level of regret you will experience **if you ...** Continued the bet but then the bus failed to arrive within the time limit

- 0 (no regret)
- 1
- 2
- 3
- 4
- 5
- 6
- 7
- 8
- 9
- 10 (very strong regret)

Q2. Estimate the level of regret you will experience **if you ...** Rejected the bet and never knew the outcome.

- 0 (no regret)
- 1
- 2
- 3
- 4
- 5
- 6
- 7
- 8
- 9
- 10 (very strong regret)

Q3. Indicate your willingness to continue the bet by giving a probability rating ranging between 0 (*absolutely no*) and 100 (*absolutely yes*).
*Reminder: The probability rating should be a number ranging between 0 to 100.*

### **Participant exclusion.**

- A total of 595 American participants were recruited online. 27 participants failed to meet the generalized exclusion criteria.
- 3 participants indicating a low proficiency of English (self-report<5, on a 1-7 scale).
- 14 participants who self-report not being serious about filling in the survey (self-report<4, on a 1-5 scale), 2 of which also indicated a low proficiency in English
- None of the participants who correctly guessed the hypothesis of this study in the funnelling section.
- 12 participants who failed the attention check (answered: 1 (completely disagree) for physically alive/ 7 (completely agree) for physically dead).
- Participants who failed to complete the survey.

**Main effects of personal responsibility and regret possibility.** A hierarchical regression analysis was conducted to examine if regret possibility and personal responsibility account for differences in escalation tendencies (refer to Table 1 for results). The findings indicated no statistically significant variance in escalation of commitment to be explained by personal responsibility (model 1, Δ*R^2^* = - 4.33e-4, p = 0.39) and regret possibility (model 2, Δ*R^2^* = - 0.01, p = 0.57). Additionally, the variance explained by the interaction of these two terms was further non-significant (model 3). These findings are inconsistent with the original study which found that regret possibility significantly improved the model fit. Overall, this study failed to find support for the main effects of personal responsibility and regret possibility in escalation situations.

| **Table 1**  Model Fit Measures | | | | | | | | | | | | | | | | |
| --- | --- | --- | --- | --- | --- | --- | --- | --- | --- | --- | --- | --- | --- | --- | --- | --- |
|  | | | | | | | | **Overall Model Test** | | | | | | | | |
| **Model** | | **R** | | **R²** | | **Adjusted R²** | | **F** | | **df1** | | **df2** | | **p** | | |
| 1 |  | 0.0365 |  | 0.00133 |  | -4.33e−4 |  | 0.754 |  | 1 |  | 566 |  | 0.385 |  |  |
| 2 |  | 0.0443 |  | 0.00196 |  | -0.00157 |  | 0.556 |  | 2 |  | 565 |  | 0.574 |  |  |
| 3 |  | 0.0447 |  | 0.00200 |  | -0.00331 |  | 0.376 |  | 3 |  | 564 |  | 0.770 |  |  |
|  | | | | | | | | | | | | | | | | |

### **Replication exploratory analysis**

#### **Anticipated regret about withdrawal and persistence**

A two-way ANOVA indicated statistically significant difference in net anticipated regret about withdrawal by regret possibility (*F*(1,564) = 10.776, *p* = .001, η*_p_*^2^ = 0.019), whereas there was no significant difference was found by personal responsibility (*F*(1,564) = 2.49, *p* = .115, η*_p_*^2^ = 0.004) and the interaction of these terms (*F*(1,564) = 0.113, *p* = .737, η*_p_*^2^ = 0.000). A Tukey post-hoc test further revealed the significant pairwise differences in net anticipated regret about withdrawal in the regret possibility and no regret possibility conditions with participants in the regret possibility conditions having higher levels of net anticipated regret about withdrawal (*t*(564) = -3.28, p = .001, *d*=-0.276).

On the other hand, a two-way ANOVA indicated statistically significant difference in anticipated regret about persistence by personal responsibility (*F*(1,564) = 19.59, *p* < .001, η*_p_*^2^ = 0.034), whereas there was no significant difference was found by regret possibility (*F*(1,564) = 0.324, *p* = .570, η*_p_*^2^ = 0.001) and the interaction of these terms (*F*(1,564) = 0.11, *p* = .741, η*_p_*^2^ = 0.000). The following Tukey post-host test indicated participants in the personal responsibility conditions having higher anticipated regret about persistence as compared to the no personal responsibility conditions (*t*(564) = -4.43, p < .001, *d*= -0.371).

Lastly, a two-way ANOVA indicated statistically significant difference in anticipated regret about withdrawal by personal responsibility (*F*(1,564) = 4.88, *p* = 0.028, η*_p_*^2^ = 0.009) and regret possibility (*F*(1,564) = 23.46, *p* < .001, η*_p_*^2^ = 0.040), however, the interaction of these terms provided no significant difference (*F*(1,564) = 0.577, *p* = .448, η*_p_*^2^ = 0.001). Tukey post-hoc analysis indicated pairwise differences in anticipated regret about withdrawal with participants in the personal responsibility conditions having higher anticipated regret about withdrawal as compared to the no personal responsibility conditions (*t*(564) = -2.21, p = .028, *d*= -0.185), and participants in the regret possibility conditions having higher levels of anticipated regret about withdrawal compared to the no regret possibility conditions (*t*(564) = -4.84, p < .001, *d*= -0.41).

For the next part of the analysis, correlation of escalation of commitment was determined with other variables such as anticipated regret about withdrawal, net anticipated regret about withdrawal, and anticipated regret about persistence. First, a moderate statistically significant positive association was found between net anticipated regret about withdrawal and escalation of commitment, *r* = 0.42, 95% CI = [0.35, 0.49], *p* < .001. Additionally, a moderate positive association was also found between anticipated regret about withdrawal and escalation of commitment, *r* = 0.44, 95% CI = [0.37, 0.50], *p* <.001. On the other hand, a small negative correlation was found between anticipated regret about persistence and escalation of commitment, *r* = -0.11, 95% CI = [-0.19, -0.03], *p* = .008.

The exploratory analysis further included a hierarchical linear regression which included all the four conditions. After controlling for the effects of age and gender, adding responsibility in Model 2 failed to make significant improvements to the model, *F*(1,510)=1.22, p=.27. On the other hand, adding the net anticipated regret about withdrawal (b= 0.42, t=10.34, p < .001) in Model 3 significantly improved the model fit, *F*(1,509)=106.81, p <.001. This is further indicated by the variances explained by the models. Model 2 accounts for approximately 0.7% variance, *F(*57,510)=1.07, p=.0346, adjusted *R^2^*= .007, whereas Model 3 accounted by approximately 18% variance, F(58,509)=3.11, p < .001, adjusted R^2^=.177. Lastly, adding anticipated regret about withdrawal (b=0.30, t = 5.54, p < .001) improved the model fit, *F*(1,508)=30.68, p < .001. Model 4 with anticipated regret about withdrawal explained 22.5% variance, F(59,508)=3.76, p < .001, adjusted R^2^=.225.

**Correlations**. As expected, net anticipated regret about withdrawal was positively associated with escalation of commitment for participants in conditions with regret possibility, *r* = 0.36, 95% CI = [0.25, 0.46], *p* < .001. A positive correlation was observed between anticipated regret about withdrawal and escalation of commitment for conditions with regret possibility, *r* = 0.39, 95% CI = [0.29, 0.49], *p* < .001. However, no significant correlation was found between anticipated regret about persistence and escalation of commitment for participants in conditions with regret possibility, *r* = -0.04, 95% CI = [-0.16, 0.07], *p* = .477.

### **Extensions**

#### **Extension 1: Further analysis for no regret possibility conditions**

To test the findings that anticipated regret would also be found in situations with no possibility of regret, Pearson’s correlation coefficient was adopted.

For the no personal responsibility- no regret possibility condition, a significant positive correlation with moderate effect was found between net anticipated regret about withdrawal and escalation of commitment, *r* = 0.40, 95% CI = [0.25, 0.53], *p* < .001. Similarly, a moderate positive correlation was also observed between anticipated regret about withdrawal and escalation of commitment for participants in the same condition, *r* = 0.51, 95% CI = [0.37, 0.62], *p* < .001. Whereas, the negative correlation between anticipated regret about persistence and escalation of commitment was statistically non-significant, *r* = -0.030, 95% CI = [-0.19, 0.14], *p =* .726.

Similarly for the personal responsibility- no regret possibility condition (condition 3), a statistically significant positive correlation with high effect was found between net anticipated regret about withdrawal and escalation of commitment, *r* = 0.56, 95% CI = [0.43, 0.66], *p* < .001. A moderate positive correlation was also found between anticipated regret about withdrawal and escalation of commitment for participants in the same condition, *r* = 0.49, 95% CI = [0.36, 0.61], *p* < .001. Lastly, a small negative significant association was found between anticipated regret about persistence and escalation of commitment, *r* = -0.33, 95% CI = [-0.47, -0.17], *p* < .001.

Overall, the findings of this part of the extension indicate that anticipated regret can also be found in conditions with no regret possibility.

**Extension: Individual differences in neuroticism**. A two-way ANOVA suggested no support for differences in levels of neuroticism amongst participants across the four conditions, *F*(1,564) = 2.62, *p* = .11, η*_p_*^2^ = 0.005, 90% CI [0, 0.018]. Additionally, the ANOVA found no support for the effect of personal responsibility, *F*(1,564) = 0.014, *p* = .91, η_p_^2^ = 0.000, 90% CI [0, 0.002] and regret possibility, *F*(1,564) = 0.003, *p* = .95, η_p_^2^ = 0.000, 90% CI [0, 0] on participant’s neuroticism.
